# Scaling-Up the Impact of Aflatoxin Research in Africa. The Role of Social Sciences

**DOI:** 10.3390/toxins10040136

**Published:** 2018-03-23

**Authors:** Francois Stepman

**Affiliations:** Platform for African-European Partnership in Agricultural Research for Development; FARA Secretariat, PMB CT 173 Cantonments, Accra, Ghana; fstepman@gmail.com

**Keywords:** food safety, aflatoxins, value chains, crop improvement, post-harvest management, maize, groundnuts

## Abstract

At the interface between agriculture and nutrition, the aflatoxin contamination of food and feed touches on agriculture, health, and trade. For more than three decades now, the problem of aflatoxin has been researched in Africa. The interest of development cooperation for aflatoxin and the support to aflatoxin mitigation projects has its ups and downs. The academic world and the development world still seem to operate in different spheres and a collaboration is still challenging due to the complexity of the contamination sources at pre-harvest and post-harvest levels. There is a growing call by research funders and development actors for the impact of solutions at a scale. The solutions to mitigate aflatoxin contamination require new ways of working together. A more prominent role is to be played by social scientists. The role of social scientists in scaling-up the impact of aflatoxin research in Africa and the proposed mitigation solutions is to ensure that awareness, advantage, affordability, and access are systematically assessed. Aflatoxin-reduced staple foods and feed would be an agricultural result with a considerable health and food safety impact.

## 1. Introduction

A considerable body of knowledge has been produced over the last three decades about the aflatoxin challenge that plagues Africa, in particular farmers, agripreneurs (agricultural small and medium businesses), and governments [1]. Since the identification of aflatoxin in feed as the cause of the death of thousands of turkey birds in the United Kingdom in the early 1960s, a huge amount of literature on aflatoxin contamination has been developed. The majority of data about mycotoxins in Sub Saharan Africa has been generated by international research institutions (like the International Agency for Cancer Research—IARC) or by organizations with development activities in Africa [2]. Additionally, “*A groan can almost be audible from scientists who work or have an interest in the field of mycotoxicology* (*fungi and mycotoxins*) *to the effect of: not another review publication on AF*” [3].

A recent paper documented the research story that has led to our current understanding of the aflatoxin contamination of grains and nuts. Over the years, increased evidence has been gathered about the role of *Aspergillus flavus* in the development of the hepatitis B virus and how it contributes to liver cancer [4]. While the world is experiencing the effects of climate change and greatly reduced crop diversity, food safety researchers and nutritionists are increasingly reporting alarming levels of fungal toxins which they might rarely have encountered in their working careers [5]. The 72nd Review of the Joint FAO/WHO Expert Committee on Food Additives [6] recorded some 28,000 publications on aflatoxin registered by the American Chemistry Society and 11,000 scientific publications in the database of PubMed. This represents almost twice the number available in the 49th Joint FAO/WHO Expert Committee on Food Additives [5,7,8].

The contamination by Aflatoxin of human food and animal feed is often occurring in African countries that have deficiencies in their storage and post-harvest handling processes. Occasionally, such contamination also occurs in Europe [9]. The climate change predictions are forecasting that many African regions will undergo periods of drought, while East Africa may experience a hotter but also more humid climate. Already, such conditions are the reason why aflatoxin thrives on the African continent more than anywhere else on the globe [10]. Depending on the general warming up of the planet, this situation might exacerbate the aflatoxin contamination crisis in Africa. Over the past 50 years, there has been a radical shift in the African diet where maize (and also rice) has taken a central place. It is precisely for this staple crop, which is cultivated in higher latitudes, that a rise in temperatures will increase the risk of aflatoxin contamination. Not only Africa will become more affected. For Europe, in the scenario of a 2 °C rise in global temperatures, aflatoxin will (again) become a food safety issue. In 2012, Serbia was affected by a heavy drought [11]. The Rapid Alert System for Food and Feed—Europe returned in 2012 maize to Serbia after reports about aflatoxin M1 in milk due to problems with differences in AFB1 limits in feed (Serbia and EU feed). According to German authorities, 10,000 metric tonnes of the contaminated feed had been delivered to 13 feed producers in Lower Saxony, which processed the maize for compound feed for cattle, pigs, and poultry. This incident prompted Germany to re-activate its national aflatoxin expertise (Kiel University, Max Rubner-Institut, Karlsruhe, Germany, etc.). The main concern of German researchers was especially the carry over effect in milk from cattle. But such alert systems are rather inexistent in developing countries. UNEP estimates that in those countries, some 4.5 million people will be exposed to mycotoxins due to poor safety and quality control of food and feed [12].

Besides maize, other crops that are typically cultivated in Africa are sensitive to moisture and fungi production which are toxic. Aflatoxins are commonly found in cereals other than maize (like rice, wheat, sorghum, and pearl millet), in oilseeds and in high concentration in the remaining cake (or waste) after the oil was pressed out of it (oil extracted from peanut, cotton, soybean, and sunflower). When aflatoxin contaminated seeds or nuts are processed into oil, the oil may be safe but the residual seed meal and cake will maintain a high concentration of the toxins. These are often used as components of livestock feeds [2]. The development of the dairy sector in many African countries is going hand in hand with the increase in commercial livestock farming. The aflatoxin contaminated feeds affect animal health leading to major economic losses by decreased performances, reproductive disorders, and increased veterinary interventions on the one hand, and on the other hand, these contaminants may impair food security by carry-over to animal products such as milk, meat, and eggs.

Africa is also producing several spices that are sensitive to proper drying and processing (chili peppers, black pepper, turmeric, ginger, coriander), and nuts originating from trees (like coconuts). Animals and humans can also be affected by mycotoxins through contaminated fruits, vegetables, products, and tissues of animal origin and through fermented foodstuff [3]. Once food or feed is contaminated along any stage of the value chain, they remain contaminated in the further stages of the chain. Due to lack of control points in Africa, there is a progressive accumulation in Aflatoxin during post-harvesting handling, transport, and processing [13,14].

This was demonstrated again in a study from Tanzania about sunflower seeds and in a study from Egypt about sugarcane. Oil extracted from sunflowers in Europe partially explains why there was a sharp decrease in the need to import peanuts from Africa to produce oil (besides being contaminated by aflatoxin). The Tanzanian researchers documented how sunflower seeds and their by-products are also at risk. There is a need to better understand the contamination of sunflower seeds and cakes related to the average consumption of sunflower seed products by humans and animals [15]. A study on sugarcane and its secondary products in Egypt detected multiple mycotoxins even when processed into juice [16]. In Egypt, sugarcane is an important economic crop with a multitude of sugarcane products. The most important are raw sugar, vinegar, and alcohol. Other derivatives are insecticides, detergents, and molasses made from sugarcane, but also chipboard, paper, and some chemicals, paints, plastics, and fiber. More research is needed to know whether mycotoxins pose a risk in these secondary products [16].

This not only calls for interventions along the whole value chain, but also for a holistic approach which involves aflatoxin experts, social scientists, and development actors on the ground. The role of social scientists in scaling-up the impact of aflatoxin research in Africa and the proposed mitigation solutions is to ensure that *awareness*, *advantage*, *affordability, and access* are systematically assessed. It will hereafter be argued that the social sciences have a critical role to play: to understand how to raise the producers’ awareness on aflatoxin contamination, what is the incentive for the farmer to adopt solutions to reduce contamination, how to ensure the affordability of these innovations, and how to provide easy and timely access to them. While these Four A’s are intuitive, few aflatoxin research findings and development initiatives or projects are able to address each of these elements systematically in a sustainable way and increase their impact.

## 2. Awareness on Aflatoxin Contamination

The cost of prevention versus the cost of the cure is not a new debate. Thirty years ago, access to medication was central in the debate to tackle the HIV/AIDS pandemic. Two schools of thought were opposing one another. The horizontal strategy advocated a general uplifting of the primary health care in Africa to tackle the high level of infections. The vertical approach convinced the international community to set up specific programs to combat HIV/AIDS, tuberculosis, and malaria. This resulted in the creation of the Global Fund to Fight AIDS, Tuberculosis, and Malaria. It took some 10 years before anti-retroviral medication was made affordable to a large population. Aflatoxin infection and its incidence on health is far more complex. The health consequences—unless acute aflatoxicosis—will reveal themselves after many years (cancer and stunting) and several co-occurring conditions may contribute to this condition.

Most agricultural extension services do not have a specific agenda which includes aflatoxins, mycotoxins, food safety, or good agricultural practices on mitigation in their messaging [17]. There is a general poor awareness about aflatoxins, let alone the dissemination of appropriate control measures to monitor contamination at the field level, during storage, and in commercialisation. Primary health care centers in Africa almost never relate liver cancer or other negative health effects to food consumption and aflatoxin [18,19]. Knowledge in most African countries is only high in areas where outbreaks have occurred in the past and this also depends on the level of education. Severe aflatoxin poisoning (aflatoxicosis) is rather exceptional, but the media have reported on death resulting from and/or the presence of aflatoxins recently in Tanzania (July 2016), Ethiopia (October 2015), Kenya, Nigeria, South Africa, Ghana, and Uganda.

Creating awareness without suggesting realistic mitigation opportunities can create panic and consumer distrust. In Ghana, a sensational newspaper heading “*Kenkey causes cancer*” (Kenkey is a national traditional dish of fermented maize porridge) said that scientific studies from major producing sites and markets in Accra have concluded comprehensively that there is a widespread occurrence of the strain that causes cancer, particularly liver cancer, in Ga kenkey [20]. This resulted in a media and consumer hysteria which had to be denounced by the government with the backup of scientists.

In 2015, in Ethiopia, a sensational press article created some embarrassment for the International Livestock Research Institute (ILRI). ILRI had published the results of its survey on aflatoxins in the Addis Ababa area and how it affected the dairy cattle feed, and as a consequence, the milk of cows. This survey showed in some of the milk samples levels of aflatoxin which were significantly higher than allowed in the European Union (EU) [21]. A consecutive article warned that ILRI did not recommend that consumers stop consuming milk and dairy products [22]. It stressed that milk has a very high nutritional value. It demonstrated clearly that the “culprit” was identified. The cause was to be found in noug cake. This cake is a by-product of noug oil which factories extract from Niger seed. The residue is used as animal feed. Possible intervention methods are the mixing with binders or more pro-actively to work with dairy feed producers to reduce or mitigate contamination in relation to their Niger seed suppliers. But the lack of nuance in the national media created panic among the consumers and triggered a considerable purchase of powder milk abroad, calling on the help from the Ethiopian diaspora. The Prime Minister of Ethiopia called on the expertise of international experts, namely Wageningen University in the Netherlands, to suggest solutions (December 2015).

In Tanzania, the number of reported victims in 2016 amounted to 12 children, but this was only the top of the iceberg. In an effort to contain the crisis, the difference in lab results, between local and international labs which were asked to confirm the high level of exposure, was not made public. In most parts of Tanzania, the knowledge of aflatoxins is low and farmers recycle aflatoxin-contaminated harvests for household consumption or as feed. The aflatoxicosis outbreak in Kenya of 2004, resulted in an obviously higher perception of risk, but has not really increased the knowledge on safety measures to minimise the exposure to aflatoxin.

Since farmers (both crop and livestock) are key stakeholders and the first target group in the aflatoxin control chain, it is commonly accepted *that priority should be given to building their capacity for adopting good agricultural practices* (*GAP*). The awareness of farmers is meant to improve the knowledge on aflatoxin control. This includes the selection of suitable varieties (of which site selection and preparation), improving post-harvest management (including sorting, drying, and the monitoring and control of moisture and rapid detection), and good storage and distribution practices. But communicating aflatoxin issues to farmers can go terribly wrong. After a long explanation on aflatoxin contamination by an expert, a farmer asked the expert: “*So, how can I help you with YOUR problem?*” [23] Farmers may also be aware of the problem but simply do not have the means to improve drying. A survey in Uganda on aflatoxin in groundnut revealed that despite the fact that 61% of households indicated that they had indeed heard of aflatoxin, 75% of households dried groundnuts on the open earth at home with only 3% using a tarpaulin, and 10% on pavement [24].

Demographic and sociologic factors also have a profound impact on aflatoxin contamination of foods. The search for employment or a different life in the cities has led to reduced workers on the farm. In reverse, the increased investments of the urban elite into agriculture and ownership of land has led to a boost in mechanized farming. This elite can afford to introduce improved varieties of grains, particularly maize. Those varieties allow for maize to be grown and harvested twice during the planting season. However, harvesting during the wet season increases the risk of harvesting aflatoxigenic fungi. The effect of mechanized harvesting on mycotoxin pollution is not controlled [3].

Awareness is a two edged sword unless the supply of good food is increased. The problem with awareness [25] is that the poorest are disadvantaged if the best nuts are sorted at the macro level [19] or at the market level [26]. Consumers will demand safe quality food only if they are aware of the health risks posed by exposure to aflatoxin-contaminated food. Presently, few pressure groups of consumers’ interests advocate on food safety issues. They would rather advocate for more transparency in mobile phone subscriptions’ price settings than support the consumer’s demand for aflatoxin-safe food. It is important to differentiate the urban consumer from the rural consumer. Farmers are the foremost biggest consumers of the maize they produce. Often, the maize of the best quality is sold into the market [27]. Therefore, the rural household consumption needs most of the incentives to change behavior. This is the approach of initiatives like Aflacontrol and AflaSTOP [20]. The introduction of drying machinery and hermetic bags tries to work on the levels of farmer awareness. The shift from traditional storing facilities to the far more effective three-layer plastic bags (distributed in a number of countries as PURDUE bags) also depends on the purchasing power.

Interventions to reduce aflatoxin contamination and the consumption of infected grain requires the involvement of socio-economists. Social scientists may set a light on how to understand behavioral change (psychology and pedagogy), resistance to change, and a better understanding of the real incentives for change and adopting new agricultural practices. Economists need to underpin this with the analysis of market incentives. Penalties or disincentives like lower prices are not really in place when farmers deliver aflatoxin-contaminated food. In addition, markets do not differentiate between aflatoxin-free and aflatoxin-contaminated food. Therefore, economists need to analyze under which conditions premium prize policies work and its detrimental effects. In the context of regional trade, competition coming from non-contaminated produce from one region may undercut the premium prize intervention in another region. Also, at an international level, it may be cheaper to import, for instance, safe maize from abroad rather than buying it at the national level at a premium prize.

Some types of waste at the level of the farm or when processing agricultural produce often end up in the plate of cattle, pigs, fish, or chicken. Moldy food and feed is frequently fed to food-producing animals. This practice is not recommended, especially given the levels of exposure linked to the consumption of the derived products such as milk which is contaminated by aflatoxin B1. To minimise financial losses and in a context of food scarcity or insecurity, it is understandable that subsistence farmers still use grains which are contaminated with aflatoxins to feed their animals.

Awareness on the adverse impact of mycotoxins should not be limited to farmers, consumers, and the professionals in the food and feed industry [3]. Courses on mycotoxins need to be embedded in the curricula for the training of agriculturists, medical personnel, and laboratory-based scientists. A public awareness campaign on the impact and prevention of mycotoxins is crucial in the present international support for the development of the agribusiness in Africa. If a country like Ethiopia wants to semi-industrialize food processing like its textile or manufacturing industry, it is imperative to overcome all the threats to food safety, in particular aflatoxin contamination.

New information products are needed based on insights from social science research on the impact of aflatoxin mitigation strategies. Some information gaps have already been identified like targeting feed processors on how aflatoxins affect animal feeds, and its consequences on animal source foods; the economic impact of aflatoxins in animal feed in Africa; cost-effective measures to manage aflatoxins in animal feed; and the impacts on human health [28].

Training videos for farmers can today be watched on mobile phone devices which have a memory chip for pictures and videos. Aflatoxin mitigation practices can thus easily reach them and many farmers can adopt them. A series of videos were for instance produced in English and Gonja, a major language in the north of Ghana where groundnut is cultivated [29]. The videos introduce aflatoxin, instruct farmers on how they can reduce contamination in their crops, and describe market opportunities for aflatoxin-safe nuts.

To support farmers in East Africa, West Africa, and Latin America, a video produced by Access Agriculture on managing aflatoxins in groundnuts has been translated into 10 local languages, specifically in Aymara (Bolivia), Bambara (Mali), Bemba (Zambia), Chichewa (Malawi), Gourmantche (spoken in Burkina Faso, northern Togo and Benin, and Niger), Hausa (Hausa is the majority language of much of northern Nigeria and the neighboring Republic of Niger), Mooré (or Mossi in Burkina Faso), Peulh Fulfuldé (Senegal, Mauritania, the Gambia, and western Mali), Quechua (Bolivia, Peru, Ecuador, Colombia and Argentina), and Zarma languages (Niger). All language versions of this video can be freely downloaded from the Access Agriculture video platform [30]. Additionally, the government of Malawi, through the Ministry of Industry, Trade and Tourism, has produced a video documentary to sensitize the public on the causes, risks, prevention, and control of aflatoxins. The documentary features champions from the public sector, consumer rights bodies, farmers’ organizations, research institutions, and other key actors [31]. The video documentary was premiered on various TV stations in Malawi.

Risk communication is only effective when accompanied by messages on possible prevention and control of aflatoxins. It is important that these be accompanied by economic studies on the costs of mitigation and social (including gender) studies on the feasibility, sustainability of the adopted solutions. Finally, awareness creation should be targeted to those populations and actors who are living in the hotspots of aflatoxin contamination, rather than a blank overall awareness campaign which does not differentiate between affected and non-affected regions and people.

## 3. Adopting a Dietary Rather than Commodity Approach

Many cereals, crops, and herbs can be affected by aflatoxins alongside the value chain of each commodity, and the risk can be multisource, leading to high levels of contamination. It is therefore recognized that food safety risks cannot only be tackled by specific commodity approaches, but also through a change in diet, or through rediscovering the traditional diverse “African diet”. Many diets in Africa contain a plethora of nutritious leafy greens. A 2015 study [32] looked at the consumption of food (both healthy and unhealthy items) and nutrients in 1990 and then again in 2010 for some 187 countries. The healthiest diets were found in Africa (nine out of the ten healthiest in the world). The then African countries ranked in descending order as best diets were Chad, Sierra Leone, Mali, Gambia, Uganda, Ghana, Côte d’Ivoire, Senegal, and Somalia.

Those results have implications for the reduction of disease and economic burdens of a poor or unsafe diet. The adoption of a more diverse diet contributes directly to the lowering of the consumption of unhealthy foods, and increases the consumption of healthier foods, or both. But this is unlikely to happen so long as the aflatoxin mitigation initiatives claiming to improve food security and food safety in Africa fail to recognize that technological fixes like hybrid maize varieties may encourage a nutrition transition or dietary habits away from healthy traditional diets and foods.

It must be acknowledged, however, that today in Africa, maize is the third most cultivated food commodity in the continent after cassava and sugar cane. Grains contribute the most to the total energy intake in Africa, with figures reaching 46%. This figure is probably higher in rural areas. At the village level, cereals and tubers and roots are the main sources of nutrition. Because these people eat large quantities of a small number of staples (e.g., maize in East Africa), they may be more at risk than people who have more diversified diets. Maize is also widely grown for animal feeds. This over dependence on grains, on which aflatoxin can thrive in rural Africa, is a major reason for the high aflatoxin load in the continent [3]. A too narrow focus on improved maize varieties which show resistance against *Aspergilus flavus* or the introduction of bio-control agents limited to maize may compound the problems caused by already high rates of undernutrition with a whole set of new diet-related health issues. When diets become less staple-based, evidence from China suggests that exposure levels and sources change [28].

The need to adopt a greater diet diversification strategy and less dependency on specific crops is also crucial for Africa’s food staples export. International regulatory agencies and major buyers, processors, and traders reject the contaminated produce before entry in key export markets. A container which leaves Dakar with low aflatoxin levels can end up in Amsterdam with heavy loads. The exporter can either choose to pay the bill for the destruction of the infested cargo in the Netherlands, have the cargo returned to Senegal, or get involved in illegal circuits and ship the contaminated commodity to a third country. When the produce that does not meet international standards is returned (in particular at a regional level), it may still enter the African food and feed value chains, leading to an increased risk of exposure to the toxins.

Standards are important barriers to trade [33]. Crop exports from Africa cannot easily access European markets due to several types of standards. Esthetic standards like size and shape may lead to considerable waste unless the national market can absorb the products which do not fit the required standard. Sanitary and phytosanitary requirements are less controversial. But a too strict and undifferentiated application can lead to big economic losses. A study of the World Bank estimated that the tightening of the Maximum Allowable Levels (MALs) of aflatoxins to four parts per billion by the EU, cost African countries $670 million in annual export losses, in particular for the export of cereals, dried fruits, and nuts [34]. Aflatoxin contamination has significantly constrained exports of peanuts from West Africa to European markets. The farmers are the biggest victims. For example, in Kenya, 2.3 million 90-kg bags were declared unfit for food or feed in 2010 due to aflatoxin contamination. In October 2011, 25 t of contaminated Unimix (a high-protein mix containing maize flour) destined for relief efforts in drought-affected areas of Kenya was recalled [35].

Brookside Dairy, established in 1993, is the largest milk processing company in East Africa, controlling about 45% of the market. The Kenyan Brookside Dairy has tried to cope with the challenges of these international standards for aflatoxin. Dairy products are an important source of Alatoxin M1 exposure because of its stability to heat, cold storage, freezing, and drying during processing [2]. Currently, the company has processing plants in both Kenya and Uganda and has devoted large areas to cultivation in order to produce safe maize feed for its milk cows with the support of drip-irrigation know-how from Israel. The Israeli Firm Green Arava was contracted to develop a 10,000-acre model farm including barns which can accommodate hundreds of milk cows and for livestock fattening. In 2015, the French food group Danone acquired a 40% stake in Brookside [36]. The Kenyatta family still remains the dominant shareholder in Brookside with a 50% stake, while Abraaj Capital, a Dubai-based private equity firm, owns 10% of the company. The five-year Galana-Kulalu irrigation project in Tana River and Kilifi was to double by 2020 the production of maize. Huge dryers were installed by the United Arab Emirates. The safe, aflatoxin-free milk is exported to Dubai. But, at the beginning of 2018, only 5000 acres, out of the targeted one million, have been put under crop.

Contrary to international milk markets, local or regional markets may be worse off. In Malawi, 60 percent of groundnuts are in markets which are poorly regulated. This exposes populations to high levels of the toxin and it undermines the food security and food safety interventions. To address the economic and health impacts of aflatoxins, it is therefore urgent to bring improvements to processing, storage, and trading practices along the smallholder groundnut supply chain [34].

Trade arguments may be more convincing for policy makers than those related to public health to enforce policies for the adoption of aflatoxin control measures in commercial markets. For this, one needs to understand the tradeoffs and the role of international standards carefully. Several aspects need to be taken into account and put into balance: realistic trade opportunities with the cost of risk reduction, and the weight of trade losses against domestic public health priorities. When standards are almost impossible to comply with and when risk reduction is too expensive, they discourage compliance, especially among poor farmers and consumers. On the other hand, “dumping” of sub-standard products is prevented when international standards are adopted.

But it would be an illusion to think that African countries can act individually. The promotion of safe regional trade is only possible if it reflects the realities of the respective national production conditions and mitigation costs. Collective adoption of regional standards is indispensable by groups of trading partners. For the majority of African countries, domestic and regional markets are much more important than export markets. Regulations have a very small effect on foods consumed on the farm or sold on informal markets, where the poorest farmers sell their products and the poorest consumers buy their products. On the contrary, a higher impact can be expected from awareness creation and intervention strategies which focus on “rediscovering” bio diversity and the nutritional qualities of a more diverse diet.

## 4. Affordability of Aflatoxin Mitigation Interventions

There are many types of interventions possible depending on whether a dietary perspective or a commodity perspective is taken. However, the affordability of aflatoxin mitigation interventions is key for upscaling. Crops which do not meet international standards lead to economic losses and eventually food waste [3]. More stringent regulations decrease the health risk factors and the cost of treatments under the public healthcare budget.

Macro socio-economic studies are needed to estimate and put into perspective the respective economic, healthcare, and environmental costs of aflatoxin mitigation strategies. Specific interventions—like innovation in cropping systems, the introduction of genetically modified variety of crops, the use of bio-control agents, the use of fungicides and pesticides, post-harvest treatments, insects control measures during storage, the propagation of new or existing fermentation techniques, and mixing animal feeds with binders—require in-depth studies to grasp the cost and affordability.

An acute need exists for methods which small-scale farmers can apply. Subsistence farmers have limited resources and they need to make rapid decisions when there is a high risk of contamination [37]. Affordability depends on the cost of good agricultural pre-harvest practices and post-harvest treatments. In October 2015 the Platform for African—European Partnership in Agricultural Research for Development (PAEPARD) published a policy paper on the role of multi-stakeholder partnerships between Africa and Europe exemplified by the issue of aflatoxin contamination of food and feed [38]. In January 2016 PAEPARD organised a Round Table of aflatoxin experts in Brussels [39]. The purpose of this aflatoxin experts meeting was to provide new perspectives, share experiences and highlight potential solutions to the contamination of food and feed with aflatoxins that are re-emerging today in Europe but since decades have been threatening livelihoods and productivity growth in Africa and many other low income countries throughout the world. In September 2016, the International Institute for Tropical Agriculture (IITA) and the International Centre of Insect Physiology and Ecology (ICIPE) were invited by the Platform for African—European Partnership in Agricultural Research for Development (PAEPARD) to suggest innovative fundamental and applied research projects which have the greatest potential to become an overall accessible solution for the mitigation of aflatoxin contamination. The push-pull system (ICIPE) is discussed in the present chapter on the affordability of aflatoxin mitigation strategies and the use of bio control agents (IITA) will be discussed in the chapter related to access to aflatoxin mitigation.

ICIPE discovered that the maize which grows in the push-pull system has less—significantly less—aflatoxin. Push-pull is a cropping system developed in the nineties where two companion plants are used. One group of companion plants attracts the stem borer (an attractive host plant like Napier grass, or Brachiaria planted as a trap plant for stem borers) and another group of companion plants repels it (desmodium). The repellent companion plant also controls striga and increases the soil fertility. Observations indicated a significantly reduced attack of maize by ear rots and mycotoxins with the push-pull technology, implying the potential contribution of the technology to food safety. This was also reported by the farmers. Given that the push-pull technology controls the stem borer, a reduction in fumonisin as well as aflatoxin is to be expected. ICIPE started to work more systematically on this aspect of research and looked into the co-relation of the stem borer attack in the second generation and aflatoxin, through the effect of desmodium on the aflatoxin in the soil.

ICIPE undertook research so that, in addition to striga control and stem borer control, the approach could also provide the farmers with aflatoxin control. Some fundamental research is needed to look into the chemicals which are produced by the root system of desmodium and their effect on the fungus in the field and to see what happens because many of those chemicals which are produced by the root system of desmodium are anti-fungal chemicals. ICIPE would like to see their effect and to know which kind of chemicals can control aflatoxin.

Fundamental research is needed to co-relate the stem borer damage with the aflatoxin incidence in the maize crops. Social and economic research is needed to understand the “push-pull” factors related to adoption rates. While good agricultural practices are much more affordable, it was expected that the adoption of the push-pull approach would have been much faster. The adoption may indeed be hindered by the labor intensity of the approach when farmers convert their land to other crops (removal of the Napier grass) and difficult access to desmodium in terms of availability (presently cultivated in few African countries) and cost.

To reduce the cost, ICIPE is therefore looking into the production of desmodium at a large scale. In collaboration with Rothamsted Research, ICIPE has so far selected Greenleaf Desmodium and Brachiaria cv Mulato, as possible component crops in the climate change-adapted push-pull technology. Brachiaria grass is gradually replacing Napier grass because it grows fast with less water and has been found to tolerate dry conditions better than Napier grass [40]. The unfortunate emergence of the fall armyworm, which has recently invaded Africa, has also caused substantial damage to maize and other crops. However, the adapted push-pull technology, developed for the control of cereal stem borers in drier agro-ecologies, is believed to be an added tool for the management of fall armyworm.

Despite the research cost, genetically modified varieties of crops may prove to be very beneficial in the long run. As a pre-harvest intervention, e.g., genetically modified (GM) Bt maize inhibits insect damage and hence fungal infection. Another type of biotechnology is the overexpression of antifungal plant defensins. Overexpression of some genes can improve the genetic resistance to *Aspergillus flavus* infection. Researchers from the International Crops Research Institute for the Semi-Arid Tropics (ICRISAT) have successfully applied this technique to peanuts. The question is whether this approach can also significantly reduce aflatoxin contamination in other crops and not just in groundnut [41].

Another important research area is research on the level of aflatoxins in traditionally processed foods in Africa (like Kenkey, fermented maize in Ghana). Applications of fermentation strategies can significantly reduce the presence of mycotoxins and their metabolites. In the fermented cereal-based foods of the EC-funded AFTER project (FP7: African Traditional Food Revisited by Research), there was no mycotoxin risk identified. But in the case of Egypt, freshly harvested wheat (not stored) is used to reduce mycotoxin levels. Much depends on details of the processing steps and whether heating is used as is in the case of the Egyptian product. Fermentation (mainly associated with lactic acid bacteria and yeast) improves the taste of food, but fermentation can also increase the availability of specific nutrients and reduce the accumulation of mycotoxins. While fermented foods have deep cultural values, their role in mitigating risk from aflatoxin contamination needs further study and technological improvements to standardise processes if they are to be promoted widely [1]. 

Finally, another post-harvest treatment is mixing animal feeds with binders. Mycotoxin binders are mainly added to compound animal feed, aiming to reduce the effects of mycotoxins on the animal, or to prevent damage by lowering the activity of mycotoxins in the gastro-intestinal tract [42,43,44]. Although the efficacy of some of these products has been demonstrated in animals, it is not known if these products can also be applied in a safe and efficient way in humans.

## 5. Access to Bio Control Agents

In consecutive and recent international conferences held on the African continent, the use of bio-control agents is proposed as a better pre-harvest tool, as the use of fungicides or chemicals (in complement to good agricultural practices) can add to production costs. Proponents of bio-control agents also suggest that breeding for disease resistant crops is time consuming and does not address the problem of emerging virulent fungal species.

For HIV/AIDS, heavily subsidized programs and an international active lobby work for lifting the patent on HIV medication have considerably reduced the price of anti-retroviral therapy. There is as yet no international consensus and lobby work to implement biological control using microbial antagonists. The research on a suitable bio control agent for the plant has the same significance as research on HIV/AIDS immunity for humans. On the curative side, the constant development of more specific anti-retroviral medication can be compared to research on finding the suitable atoxigenic strains with the greatest impact considering the high diversity of African soils.

Biological control using the microbial antagonist strategy has emerged as a promising approach for the control of pre-harvest contamination of aflatoxins. The antagonist microorganisms include competitive atoxigenic strains of yeasts or bacteria, and symbiotic fungi. In Africa, some microorganisms, almost exclusively atoxigenic strains of *Aspergillus* spp., are already available as branded products. Apart from a number of scientific challenges which are still being addressed (the biocontrol strain could increase the aflatoxin contamination problem through vegetative fusion and sexual reproduction), the affordability of products like aflasafe by poor farmers is difficult to resolve.

In-depth financial and economic studies have been commissioned by the International Institute for Tropical Agriculture (IITA) for the commercialization of aflasafe and releasing non-aflatoxigenic strains of *Aspergillus flavus* into the agricultural environment. This results in the suppression of naturally occurring aflatoxigenic strains [45,46]. While it seems logical that prevention (the contamination at soil level) is better than the cure, the cost of cure and prevention are difficult to estimate in view of the short, middle, and long term impact. Post-harvest interventions may be cheaper in the short run, but is not dealing with the initial cause of the contamination prevalent in the soil. We discuss the use of bio-control agents (which is sprayed on the fields) in the next chapter on access to mitigation.

IITA has been pioneering the Aflasafe Technology Transfer and Commercialisation Project (ATTC). This project works with private companies or government entities to ensure Aflasafe products reach millions of farmers. The following countries are targeted: (a) in East Africa: Kenya, Uganda, and Tanzania; (b) in West Africa: Senegal, The Gambia, Burkina Faso, Ghana, and Nigeria; and (c) in Southern Africa: Mozambique, Malawi and Zambia. However, funding constraints have limited the spread of the program to additional Sub-Saharan Africa countries.

In Kenya, Aflasafe is presently being used on maize. The Kenya Agricultural and Livestock Research Organisation (KALRO) has built an Aflasafe factory to manufacture it locally and Kenya’s Ministry of Agriculture is making it available to vulnerable farmers in areas with high aflatoxin prevalence (like Machakos). In the near future, this program may be extended to groundnuts and sorghum crops.

Despite the breakthroughs, however, the implementation of the ATTC project in Kenya has faced challenges. In late 2016 to early 2017, Kenya was affected by a long drought that led to postponing the use of Aflasafe until the next sowing season. The use of aflasafe with efficacy can also be problematic when farmers do not adhere to the correct product instructions. Another limitation has been improper timing of the stage of application of Aflasafe. For illiterate farmers, it is difficult to adhere to strict timelines. Extension agents need to provide continuous training and reminders plus monitoring. Finally, because sorghum is a key raw material in Aflasafe production, the cost of sorghum has an effect on the price of Aflasafe and may not be justifiable in times of drought and hunger.

Since 2007, there has been increased competition for sorghum because sorghum has an important industrial application: it is used more and more for beer brewing. As a consequence, sorghum production in Africa has increased significantly and is favored more and more by farmers who used to produce rice and wheat [7,47]. The renewed focus on sorghum is also because it is one of the most drought-tolerable crops and such high water-use efficient characteristics make it the crop of choice to boast food security in drought-stricken regions of Africa and for the future against the anticipated water scarcity in the world. Despite the increasing economic value of sorghum, it is rather worrying that there is limited research on mycotoxins in sorghum produced in Africa [3].

Access to a bio control solution can easily take more than a decade between the initial research and rolling out a solution. It took Italy more than 10 years to carry out the first large-scale trial application of an *Aspergillus flavus* biocontrol agent for aflatoxin prevention in Italian maize. This is critical for the north of Italy, which produces world renowned cheese and other dairy products. Local Italian genotypes of *A. flavus* have been collected since 2003 [48]. The identified bio control agent proved to be very effective at preventing aflatoxin contamination of Italian maize, which is an important feed resource for cattle. The farming industries have urged the registration process of this bio control agent to be speeded up in order for them to be able to spray this agent on their field and guarantee food and feed safety in the maize and dairy value chains. The long time gap is partly due to the present way of funding mycotoxin research (short term research funding and funding interruptions) and the necessary clearances by the national food agency.

The scientific community has also expressed its concern that actions that are effective for reducing aflatoxin in maize and do not reduce fumonisin exposure may not result in a reduced cancer burden and would not reduce, for example, the risk of birth defects from fumonisin exposure [49]. The evidence supports actions that concurrently reduce the amounts of both toxins in maize versus actions that reduce aflatoxin alone. South Africa reported the structure of fumonisin co-concurrently with some knowledge of its effects over 25 years. There is now adequate evidence that fumonisin exposure in the early stages of pregnancy results in birth defects in folate deficient populations [50]. Decision-makers need to be briefed on the fact that difficult choices are now inescapable. *How will actions that mainly address population level exposure to aflatoxin in maize also exposed to fumonisin be regarded 10 years from now*? [8].

While the bio-control agent aflasafe is receiving considerable external and sustained support to be rolled out in the field and increase the access to farmers through its mass production, this should not exclude close monitoring of the performance of Aflasafe products from researchers not connected to IITA.

## 6. Access to Rapid Diagnosis

The Partnership for Aflatoxin Control in Africa (PACA) in its rolling out of aflatoxin mitigation strategies is challenged by the lack of political commitment, poor infrastructure, unreliable or discontinued distribution systems, and stringent market mechanisms similar to the challenges which confronted the interventions during the HIV/AIDS pandemic. The detection of HIV/AIDS became much cheaper with the general use of the Elisa test, which was later on followed up by more expensive testing like CD4 cell count testing. Few laboratories could initially perform such testing. Rapid test kits can follow a similar path of development. The type of equipment needed for the confirmation of the level of mycotoxins contamination in the laboratory is very costly and therefore only accessible to few researchers who often do not have national facilities to test their samples. Aflatoxin researchers have to travel to another African country (like the ILRI/BECA hub in Nairobi) or send their samples abroad to cope with the lack of trained laboratory personnel able to use the test equipment, or because the testing supplies are too expensive due to the need of regular maintenance and repairs of the instrument. Many African labs cannot guarantee the quality control and assurance schemes.

The development of rapid diagnostics and new control technologies through research was the object of a PACA-GIZ study [51]. The importance of affordable, accessible, and accurate rapid test kits for tests at all critical points of the value chain cannot be overestimated. It can incentivize different value chain actors in the local markets and reward aflatoxin control. The provision of affordable and accessible rapid test kits for tests at all critical points of the value chain (VC) directly benefits risk communication and motivate the changes in practices [52]. A confirmation of critical control point where most contamination occurs (example during long transports) can inform simple risk reduction measures (like ventilation to reduce the moisture levels). The PACA-GIZ study demonstrates that cost is closely linked to the purpose of the test: do the results need to be quantitative or qualitative? For complex, equipment intensive, and operator sensitive tests, an appropriate laboratory environment is needed which can be very expensive as an initial investment, but also in the maintenance of the laboratory equipment and reagents. Besides the cost of testing, the other considerations which need to be taken into account are: easy handling like portable devices, low expenditure at time, the possibility of sensitive and multiple analysis, the exclusion of false positive results, and the need of trained (scientific) staff, etc.

The PACA-GIZ study was complemented by a “public” survey in the framework of the research activities of the Mycokey project: “Integrated and innovative key actions for mycotoxin management in the food and feed chain”. This survey was launched in March 2018 to share current experience and future needs on rapid methods for mycotoxin screening. The overall objective was to promote interactive discussions between scientists involved in rapid methods developments within the MycoKey and customers/stakeholders to deliver new and validated rapid methods that fit stakeholder needs and expectations.

The availability of affordable rapid diagnosis test kits should dramatically increase the availability of data [53]. This can generate an unprecedented set of data on aflatoxin (and more generally mycotoxin) incidence across commodities and countries in sub-Saharan Africa. [54] Using a standardized set of procedures and protocols the assembly of these data will enable meta-analysis. Aflatoxin surveys and studies on the continent have often used diverse procedures for sampling and analysis, and as such, meta-analysis across them is not easy. The type of data presently available from (often fragmented) research projects spans maize, rice, milk and feed, groundnuts and tree nuts, etc. It includes information on aflatoxin and other toxin levels in different food and feed commodities; the morphological, biochemical and genetic characterization of aflatoxin-producing fungi from the same; aflatoxin resistance levels of different popular crop (in particular maize) varieties and breeding lines; and information about farmer practices, the crop type grown and toxin levels (again mainly researched for maize). 

ICT, communication experts and (young) app developers can play a crucial role here. Statistically rigorous analytical models and visualization tools can explore the likely impacts of aflatoxin mitigation interventions at different spatio-temporal scales. Those tools can be used to identify potential location-specific management options to reduce aflatoxin risks in and provide a baseline map of the aflatoxin risks. With rapid and affordable test kits, risks can be monitored and updated through time, capturing the inter-annual variation in risks and the impacts of interventions. This allows for an evolution into a dynamic predictive models by the assimilation of ongoing monitoring, by sensor network, by extension and researchers using rapid aflatoxin tests kits, and by the use of other data sources. These data can form the basis upon which information and awareness creation tools can be extracted, to inform and populate for instance mobile phone apps and videos. Prediction models and maps can be used to identify areas where sensors and apps should be targeted for deployment: e.g., within identified historical hotspots, with potential cross-reference to places where climatic conditions raise postharvest storage risks in a way that can be mitigated with specific interventions, and where farmers have access to markets and extension to get interventions if they receive messages/information via a mobile phone app. 

But those apps need to be backed by sound data so that maps, models and other outputs can reach an expanded set of farmers and stakeholders. Extension officers and others need to receive the information during harvest and after harvest, so that they can identify the right sampling protocol when encountering a contamination scenario (farm store vs posho mill, with different sizes of maize stored, etc.). Apps can also potentially be designed to deliver subsidies for interventions related to the driver of emerging aflatoxin risk in hotspot areas (e.g., insect pest outbreaks, rainfall around harvest time that will complicate drying, etc.). During an outbreak, such information and the apps drawing upon it could help a significant number of smallholder farmer families. The geographical risk models can help inform the development, deployment and testing of strategic intervention techniques in a timely, geographically targeted manner for delivery of time sensitive information on risks.

## 7. Conclusions

The concern for overall food safety and aflatoxin contamination in Africa should not only remain the responsibility of mycotoxin experts. There is an urgent need to engage social scientists to assess the awareness, advantage, affordability, and access of mitigation solutions.

The levels of contamination of foods and feeds in Africa which commonly exceed internationally acceptable standards prompt the need to urgently upscale strategies. No country is immune and African consumers and livestock remain at risk. The role of social scientists in scaling-up the impact of aflatoxin research in Africa and the proposed mitigation solutions is to ensure that awareness, advantage, affordability, and access are systematically assessed. Social science research is needed to help researchers who are developing further aflatoxin control strategies to better position their interventions among various existing strategies in terms of economic feasibility.

Consumers also need to be made more aware of the health risks posed by exposure to aflatoxin-contaminated food and to be empowered to demand safe, quality food. Awareness creation and intervention strategies should also focus on “rediscovering” bio diversity and the nutritional qualities of a more diverse diet and traditional African diet. The best interventions to outright prevent acute toxicity relate to improving dietary diversity. Awareness campaigns are to go hand in hand with realistic mitigation opportunities which can be up-scaled at an affordable cost. New information products are needed based on additional aflatoxin research and the impact of mitigation strategies from social science research. Risk communication includes information about simple risk reduction measures. Food safety risks need to be addressed using a dietary rather than a commodity perspective.

There are many types of interventions possible depending on whether a dietary perspective or a commodity perspective is taken. However, the affordability of aflatoxin mitigation interventions is key for upscaling. While good agricultural practices are much more affordable, accessible, and easier to upscale, biological control using the microbial antagonist strategy has emerged as a promising approach for the control of pre-harvest contamination of aflatoxins. This paper highlights, however, that bio control solutions are faced by challenges. From the perspective of affordability and access, the bio control solution can easily take too long between the initial research and rolling out of a solution. While mass production of a bio control agent is a lofty endeavor to reduce the cost, its roll out requires considerable investments in production facilities, market availability, awareness campaigns, and vulgarisation on how it works, and assuring a competitive advantage through premium price policies.

The need to access affordable (cheap) food in an environment of acute food scarcity and insecurity hinders the impact of a lot of well-intentioned interventions and strategies to guarantee safe food with acceptable levels of aflatoxin. The concerns for food safety and healthy food depends on the level of GDP. The expected economic growth of Africa justifies increased investments to upscale mitigation solutions and strategies. A balance must be found between pre-harvest interventions which tackle the root causes of the contamination and the profound impact of good agricultural practices and post-harvest actions.
